# Introducing higher harmonic generation microscopy for renal tumour pathology: a perspective on clinical translation

**DOI:** 10.1007/s12551-026-01453-8

**Published:** 2026-09-10

**Authors:** M. Duijn, S. Spies, L. T. de Haan, D. M. de Bruin, M. M. Lange, C. D. Savci, O. M. van Delden, M. L. Groot, P. J. Zondervan

**Affiliations:** 1 Department of Urology, Amsterdam University Medical Centers, University of Amsterdam, PO Box 7057, 1007 MB Amsterdam, The Netherlands; 2 Imaging and Biomarkers, Cancer Center Amsterdam, Amsterdam, The Netherlands; 3 LaserLab Amsterdam, Department of Physics, Faculty of Science, Vrije Universiteit Amsterdam, Amsterdam, The Netherlands; 4 Department of Biomedical Engineering and Physics, Amsterdam University Medical Centers, University of Amsterdam, Amsterdam, The Netherlands; 5 Department of Pathology, Amsterdam University Medical Centers, University of Amsterdam, Amsterdam, The Netherlands; 6 Department of Radiology and Nuclear Medicine, Amsterdam University Medical Centers, University of Amsterdam, Amsterdam, The Netherlands

**Keywords:** Renal cell carcinoma, Higher harmonic generation microscopy, Haematoxylin and eosin histology, Second harmonic generation, Third harmonic generation, Multiphoton autofluorescence, Label-free imaging, Digital pathology, Optical biopsy

## Abstract

Higher harmonic generation microscopy (HHGM) is an emerging nonlinear optical imaging modality that combines second harmonic generation (SHG), third harmonic generation (THG), and two-photon excited autofluorescence (2PEF) to generate high-resolution, label-free images of fresh biological tissue. By exploiting intrinsic optical contrast arising from nonlinear light–tissue interactions, HHGM enables rapid visualisation of cellular morphology, extracellular matrix architecture, and the tumour microenvironment without fixation or staining. These unique biophysical properties have facilitated the clinical translation of HHGM for real-time tissue assessment across several oncological applications. Renal tumour pathology represents a promising yet underexplored application of HHGM, particularly because rapid assessment of biopsy adequacy and tumour representativeness remains an important unmet clinical need. In this perspective, we discuss the biophysical principles of HHGM, review the current evidence supporting its application in oncological pathology, and present representative images that illustrate the characteristic multimodal appearance of renal cell carcinoma (RCC) and normal renal parenchyma, highlighting qualitative correspondence with conventional haematoxylin and eosin histology. In addition, HHGM enables visualisation of stromal architecture and the tumour microenvironment in fresh, unprocessed tissue within minutes. Although the ongoing prospective Intelligence Imaging (INTIMA) study is designed to validate the diagnostic accuracy, reproducibility, and clinical utility of HHGM in renal tumour pathology, these findings should be considered preliminary until such validation is completed. If confirmed, together with continued advances in nonlinear optical microscopy, digital pathology, and artificial intelligence (AI), HHGM could become a valuable adjunct to routine histopathological assessment and real-time biopsy evaluation.

## Introduction

Higher harmonic generation microscopy (HHGM) is an emerging label-free imaging modality that combines second harmonic generation (SHG), third harmonic generation (THG), and two-photon excited autofluorescence (2PEF) to generate high-resolution images of fresh biological tissue without fixation, sectioning, or exogenous staining (Campagnol et al. 2003; Débarre et al. 2006; Cox 2011). By exploiting intrinsic nonlinear light–tissue interactions, HHGM provides endogenous contrast from tissue structures such as collagen, lipids, and cellular interfaces (Gorrel et al. 2003; Provenzano et al. 2008; Kuzmin et al. 2016), enabling rapid, histology-like visualisation of fresh, unprocessed tissue (van Huizen et al. 2025; Spies et al. 2025). These unique characteristics have generated increasing interest in HHGM as a rapid optical pathology technique.

Recent advances in ultrafast laser technology and compact imaging systems have accelerated the translation of HHGM from experimental research to clinical pathology. Feasibility studies have demonstrated high concordance with conventional haematoxylin and eosin (H&E) histology in several applications, including lung and paediatric renal tumour pathology, while enabling rapid assessment of tissue adequacy immediately after biopsy or surgical excision (van Huizen et al. 2025; Spies et al. 2025). Together with complementary label-free imaging techniques such as stimulated Raman scattering microscopy, these developments reflect the broader evolution of real-time optical pathology (Orringer et al. 2017; Groot and Spies 2025).

Renal cell carcinoma (RCC) represents an attractive application for HHGM. Percutaneous renal tumour biopsy plays an increasingly important role in the diagnostic work-up of small renal masses and before minimally invasive treatments such as thermal ablation and stereotactic ablative body radiotherapy. However, non-diagnostic biopsy rates of approximately 10–20% remain a clinically relevant challenge, frequently necessitating repeat procedures and delaying treatment. A rapid imaging modality capable of confirming biopsy adequacy and tumour representativeness at the point of care could therefore improve current diagnostic pathways. In addition, RCC exhibits considerable histological and microenvironmental heterogeneity, characterised by variable stromal composition, tumour vascularity, immune cell infiltration, and diverse histological subtypes. These features make RCC well suited to multimodal nonlinear imaging, as SHG, THG, and 2PEF provide complementary information on extracellular matrix organisation, cellular morphology, and endogenous tissue contrast.

In this perspective, we review the biophysical principles of HHGM, summarise the current evidence supporting its application in oncological pathology, and discuss recent advances towards its clinical translation. Finally, we discuss the opportunities and remaining challenges for integrating HHGM into routine renal tumour diagnostics.

## Biophysical principles and imaging workflow

**Fig. 1:**
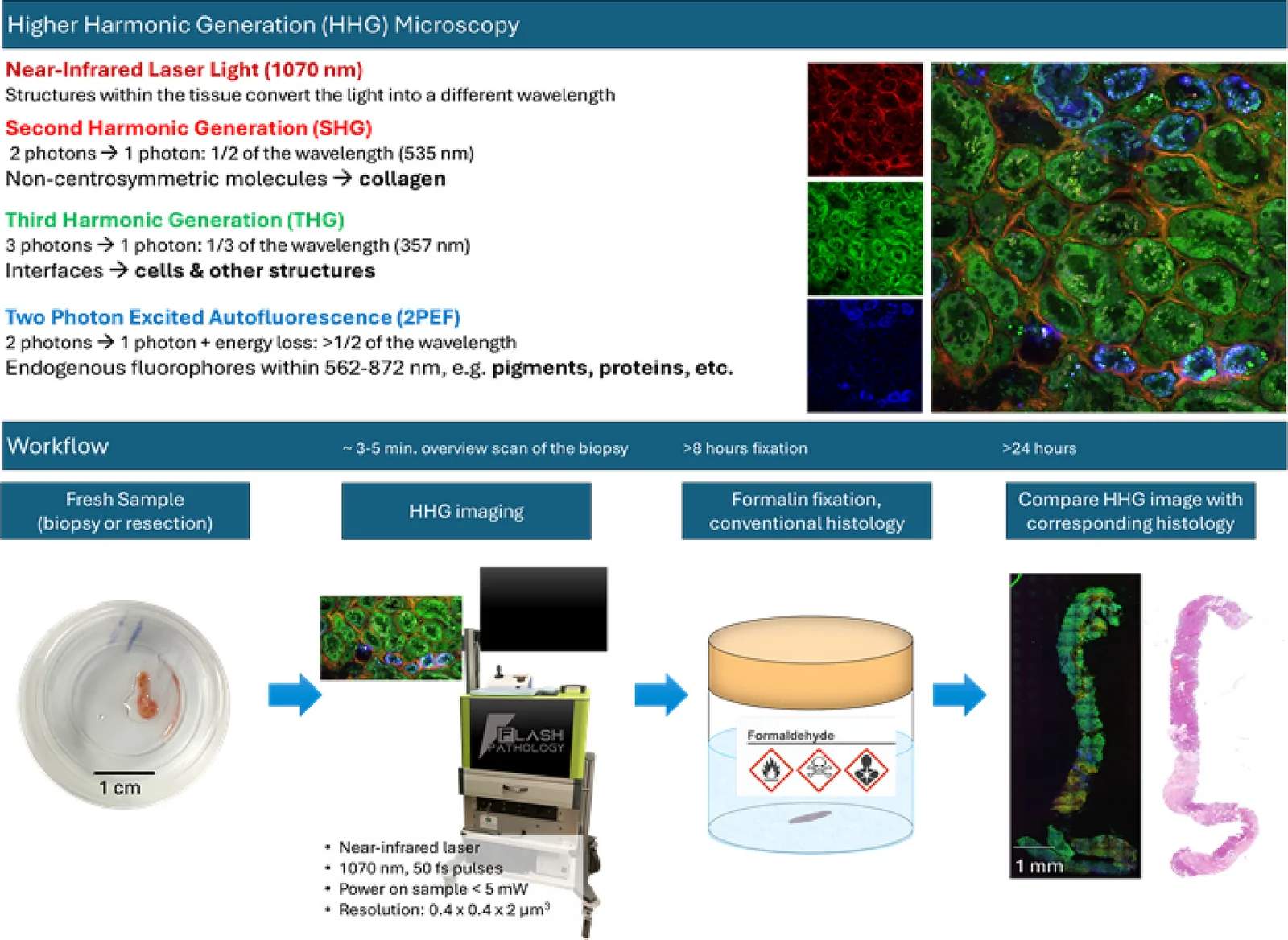
Clinical workflow of higher harmonic generation (HHG) microscopy. HHG microscopy combines third harmonic generation (THG), second harmonic generation (SHG), and two-photon excited autofluorescence (2PEF) to generate label-free, high-resolution images of fresh biological tissue without fixation, sectioning, or staining. Fresh biopsy or surgical specimens are placed in the microscope immediately after acquisition and scanned using an automated mosaic acquisition. An initial low-resolution overview scan identifies the region of interest, followed by high-resolution imaging of selected areas. Because HHG imaging is non-destructive, the tissue remains intact after imaging and is subsequently processed according to the routine histopathology workflow, including formalin fixation, paraffin embedding, sectioning, and hematoxylin and eosin (H&E) staining, allowing direct comparison between HHG and conventional histology

Successful clinical implementation of HHGM depends on standardised imaging instrumentation, tissue handling, and image acquisition protocols. Although technical configurations differ between research groups, the overall workflow is largely consistent and has already been translated to several clinical applications, including lung and paediatric renal tumour pathology (van Huizen et al. 2025; Spies et al. 2025). The general HHGM workflow is illustrated in Fig. 1.

HHGM employs ultrafast femtosecond near-infrared laser pulses to generate SHG, THG, and 2PEF. These endogenous contrast mechanisms provide complementary information on tissue architecture and composition (Cox 2011; Gorrell et al. 2003; Provenzano et al. 2008; Kuzmin et al. 2016) and can be combined into pseudo-colour images that closely resemble conventional H&E histology (van Huizen et al. 2025; Spies et al. 2025). Because image contrast is generated intrinsically, fresh tissue can be imaged without fixation, sectioning, staining, or exogenous contrast agents, allowing subsequent routine histopathological processing and additional tissue analyses (van Huizen et al. 2025; Spies et al. 2025; Groot and Spies 2025).

Following biopsy or surgical resection, fresh tissue is placed directly onto a glass imaging surface and scanned to identify regions of interest for high-resolution imaging. Image acquisition typically requires only 3–5 min per specimen, enabling rapid assessment while preserving tissue integrity. Because HHGM is performed on fresh, unstained tissue without altering the specimen, the same sample can subsequently undergo standard formalin fixation, paraffin embedding, sectioning, and H&E staining. Importantly, the preserved tissue also remains available for additional histopathological or immunohistochemical staining when clinically indicated.

Recent advances in compact fibre-laser technology, automated image acquisition, and digital image analysis have improved the feasibility of integrating HHGM into routine pathology workflows. Furthermore, the inherently digital nature of HHGM datasets facilitates quantitative image analysis and future integration with artificial intelligence (AI)-based diagnostic algorithms.

Despite these advances, widespread clinical implementation will require further reductions in system complexity and cost, together with standardisation of imaging protocols, quality assurance procedures, and image interpretation criteria. Continued technological developments are expected to improve robustness, accessibility, and scalability for routine diagnostic practice.

## Clinical translation: current evidence in optical pathology

The past decade has witnessed rapid progress in the clinical translation of nonlinear optical microscopy from experimental research to real-time diagnostic pathology. Early studies established the feasibility of SHG and THG for visualising tissue architecture and extracellular matrix organisation (Campagnola et al. 2003; Débarre et al. 2006; Cox 2011; Gorrell et al. 2003; Provenzano et al. 2008; Kuzmin et al. 2016), while more recent work has demonstrated that multimodal HHGM can generate histology-like images from fresh tissue within minutes (van Huizen et al. 2025; Spies et al. 2025). Collectively, these advances have laid the foundation for the clinical implementation of HHGM across multiple oncological applications.

One of the first successful clinical applications of HHGM was reported in pulmonary pathology, where fresh lung and pleural biopsy specimens were imaged immediately after tissue acquisition. In a study of 109 biopsies from 47 patients, a portable HHGM system generated a biopsy overview image within a median of 6 min after excision, providing histological information from unprocessed tissue at a time point when conventional histopathology is not yet available. Image quality was sufficient for malignancy or non-malignancy assessment in 97% of biopsies, and 87% of HHG images were correctly scored by pathologists. The combination of rapid acquisition, unprocessed tissue imaging, and AI-assisted assessment demonstrates the potential of HHGM to provide immediate pathological feedback at the point of biopsy, rather than requiring conventional tissue processing before histological evaluation (van Huizen et al. 2025). Similarly, HHGM has recently been evaluated in paediatric renal tumours in a prospective multi-observer study. Experienced pathologists achieved high diagnostic accuracy in distinguishing tumour from normal renal tissue and in assessing specimen representativeness using HHGM alone, demonstrating that the technique reliably reproduces diagnostically relevant morphological features while substantially reducing the time to pathological assessment (Spies et al. 2025).

Beyond HHGM, other nonlinear optical imaging modalities further support the concept of real-time optical pathology. Stimulated Raman scattering microscopy, for example, has demonstrated diagnostic performance comparable to conventional histopathology during intraoperative assessment of brain tumours and other malignancies, illustrating the broader transition towards rapid, label-free optical diagnosis (Orringer et al. 2017; Groot and Spies 2025).

**Fig. 2:**
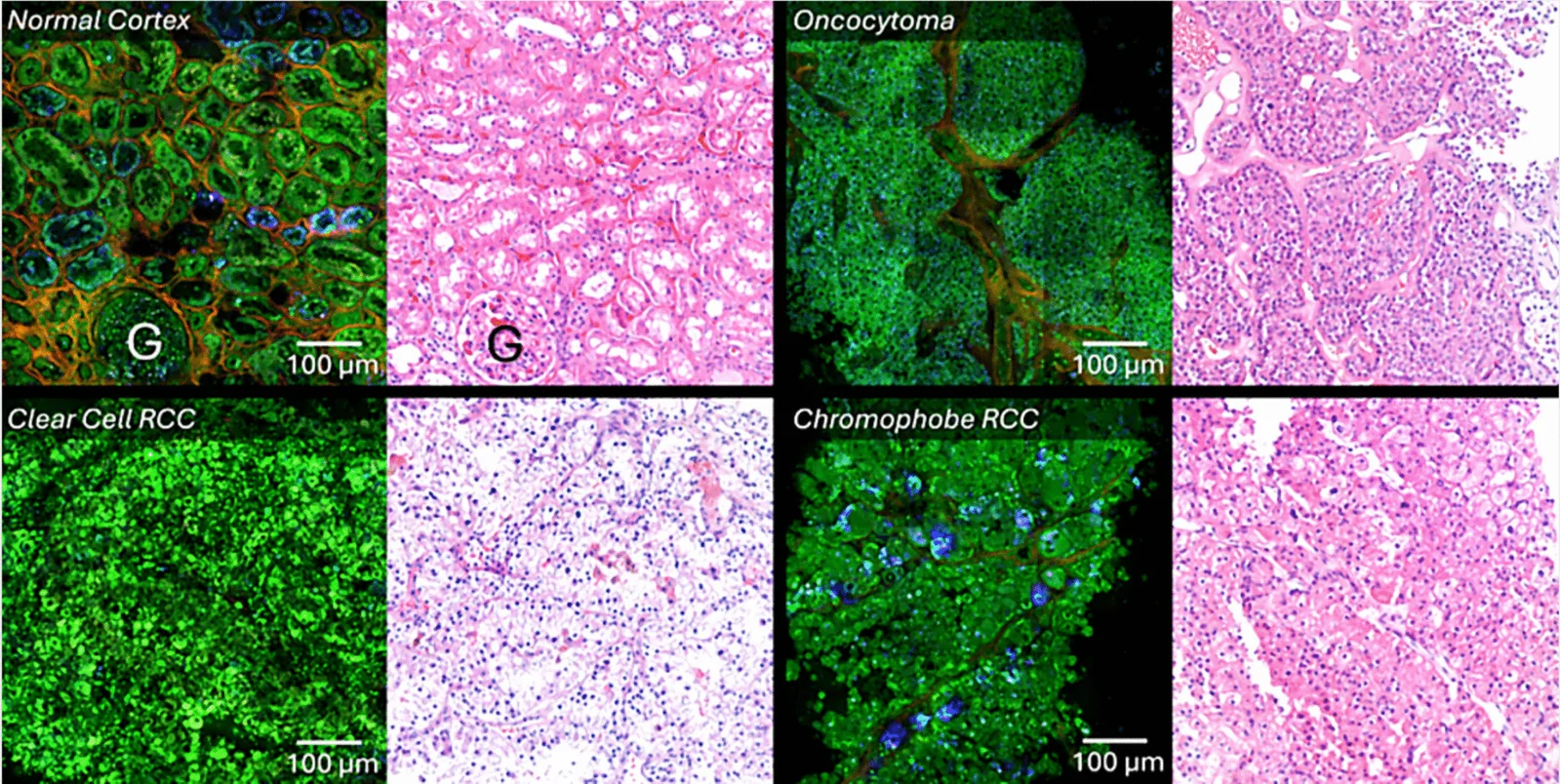
Representative higher harmonic generation microscopy (HHGM) images compared with corresponding hematoxylin and eosin (H&E) histology. HHG (THG/SHG/2PEF) imaging vs. conventional histology (hematoxylin and eosin, H&E). HHG visualises cells, collagen, and autofluorescence without staining. Normal cortex shows tubules and glomeruli (G) with surrounding collagen; nuclei appear as dark cytoplasmic voids. Tumours display loss of normal renal architecture. Oncocytoma forms small collagen-surrounded nests with granular cytoplasm. Clear cell RCC shows bright, granular cells due to cytoplasmic lipid droplets (clear in H&E). Chromophobe RCC presents sheets of large polygonal cells with smaller interspersed cells. Images are representative and intended to illustrate characteristic HHGM features rather than diagnostic performance

Although clinical experience in adult RCC remains limited, the morphological heterogeneity of RCC makes it a particularly attractive application for multimodal nonlinear imaging. The complementary information generated by SHG, THG, and 2PEF could enable simultaneous assessment of tumour architecture and stromal composition, potentially providing quantitative features beyond conventional morphological assessment. Representative HHGM images of normal renal cortex and common renal tumour subtypes are shown in Fig. 2, serving to illustrate the feasibility of this multimodal approach and its qualitative relationship with conventional H&E histology rather than to demonstrate diagnostic performance.

## Future perspectives and remaining challenges

HHGM is emerging as a promising addition to the expanding field of optical pathology. By combining SHG, THG, and 2PEF, HHGM generates high-resolution, histology-like images of fresh tissue without fixation, sectioning, or staining. This unique combination of rapid image acquisition, endogenous contrast, and preservation of tissue for downstream histopathological and molecular analyses distinguishes HHGM from conventional pathology workflows and highlights its potential as an adjunctive diagnostic technique rather than a replacement for routine histopathology. Importantly, the complementary contrast mechanisms underlying HHGM provide information that cannot be obtained from a single imaging modality alone. SHG selectively visualises non-centrosymmetric structures such as fibrillar collagen, enabling assessment of extracellular matrix organisation, whereas THG is generated at refractive index discontinuities, highlighting cellular membranes, intracellular interfaces, and lipid-rich structures. Simultaneously, 2PEF detects endogenous fluorophores, including NADH, FAD, elastin, and lipofuscin, providing additional functional and morphological information. The integration of these nonlinear optical signals into a single pseudo-colour image contributes to the close resemblance between HHGM and conventional H&E histology.

The successful clinical translation of HHGM in lung and paediatric renal tumour pathology demonstrates that the technique can reproduce diagnostically relevant tissue morphology while enabling rapid assessment of biopsy adequacy (van Huizen et al. 2025; Spies et al. 2025). These studies support the broader concept of real-time optical pathology, in which label-free imaging complements conventional histopathology by providing immediate information at the point of tissue acquisition. Together with advances in stimulated Raman scattering microscopy and other nonlinear optical imaging techniques, these findings illustrate the growing clinical potential of optical pathology across multiple oncological applications (van Huizen et al. 2025; Spies et al. 2025; Orringer et al. 2017; Groot and Spies 2025). Although these technologies exploit different contrast mechanisms, they share the common objective of providing rapid, label-free tissue characterisation while reducing dependence on conventional tissue processing. In this context, HHGM offers the practical advantage of simultaneously visualising cellular morphology, stromal architecture, and extracellular matrix organisation.

RCC represents a particularly attractive indication for HHGM because timely confirmation of biopsy adequacy could reduce non-diagnostic sampling, minimise repeat procedures, and facilitate earlier treatment decisions. Furthermore, the heterogeneous architecture of RCC, including its stromal composition, vascular network, and diverse histological subtypes, is well suited to multimodal nonlinear imaging. The representative images shown in Fig. 2 illustrate the characteristic multimodal appearance of normal renal cortex and common RCC subtypes, demonstrating the ability of HHGM to visualise tissue architecture, collagen organisation, and cellular morphology using endogenous optical contrast. Moreover, the lipid-rich cytoplasm of clear cell RCC, abundant stromal collagen in selected tumour subtypes, and complex tumour–stroma interactions generate distinct nonlinear optical signatures, suggesting that HHGM may eventually facilitate quantitative assessment of tissue composition beyond conventional qualitative morphology.

Beyond conventional morphology, HHGM may provide additional opportunities for assessing the tumour microenvironment. In particular, SHG imaging can enable quantitative characterisation of stromal collagen, including fibre density, length, width, orientation, alignment, straightness or curvature, and dispersion, as well as the spatial organisation of fibres relative to the tumour–stroma interface. These features can capture distinct aspects of extracellular matrix remodelling that are not readily assessed by conventional H&E morphology alone. Across several solid tumours, increased collagen density and more organised or aligned fibre architectures have been associated with tumour progression, metastatic potential, treatment response, and clinical outcomes, although the prognostic relevance of individual collagen parameters varies between tumour types. In RCC, whether these collagen-derived imaging features can characterise tumour–stroma interactions or provide prognostic or predictive information remains unknown and warrants prospective investigation. Future studies combining HHGM with computational image analysis may enable extraction of standardised quantitative collagen biomarkers, such as fibre orientation and alignment, collagen density, fibre morphology, and spatial heterogeneity, potentially providing additional information beyond routine histopathological evaluation. Compared with other emerging optical imaging modalities, HHGM offers several practical advantages, including multimodal endogenous contrast, preservation of tissue for routine histopathology and molecular analyses, and compatibility with digital pathology workflows. Nevertheless, important challenges remain before widespread clinical implementation can be achieved. Current systems require specialised instrumentation, dedicated image-processing software, and standardised acquisition protocols. In addition, current HHGM systems are associated with relatively high equipment costs, require ultrafast laser technology and specialised optical expertise, and remain available primarily in research settings. Technical limitations, including a restricted field of view, limited imaging depth inherent to nonlinear optical microscopy, and the need for sequential image mosaicking for larger specimens, may also affect workflow efficiency. Furthermore, image quality may be influenced by tissue orientation, optical scattering properties, and inter-platform variability, emphasising the need for standardised acquisition protocols and robust quality assurance measures. Continued technological advances, together with harmonisation of imaging procedures and pathologist training, will be essential for broader clinical adoption.

Several limitations should be acknowledged. First, the representative images included in this Perspective are intended to illustrate the imaging characteristics of HHGM and do not provide quantitative evidence of diagnostic performance. Second, prospective validation in adult RCC is still lacking. Ongoing studies are evaluating the diagnostic accuracy, reproducibility, interobserver agreement, and clinical utility of HHGM, which will determine whether the promising qualitative observations reported to date translate into clinically meaningful improvements in renal tumour diagnostics. Furthermore, standardised image interpretation criteria and reproducibility across different imaging platforms and institutions remain to be established before routine implementation can be considered.

Finally, integration of HHGM with AI and digital pathology platforms represents an important future direction. Automated image analysis may improve diagnostic consistency, facilitate real-time assessment of biopsy adequacy, and support clinical decision-making within digital pathology workflows (van Huizen et al. 2025; Bera et al. 2019; Aumann et al. 2019). Beyond assisting visual interpretation, AI may enable automated tissue segmentation, tumour subtype classification, and extraction of quantitative optical biomarkers from multimodal HHGM datasets, potentially reducing observer variability and expanding the diagnostic utility of the technique. Such developments could position HHGM as a valuable complementary technology within modern pathology practice.

In conclusion, HHGM represents a promising label-free imaging modality with considerable potential to complement conventional renal tumour pathology. Its ability to generate rapid, multimodal images of fresh tissue while preserving specimens for routine histopathological, immunohistochemical, and molecular analyses makes it well suited for future applications in renal tumour diagnostics. Prospective clinical validation remains essential and is currently being addressed by the ongoing Intelligence Imaging (INTIMA) study, a prospective clinical study specifically designed to evaluate the diagnostic accuracy, reproducibility, interobserver agreement, and clinical utility of HHGM in renal tumour biopsies. Together with continued advances in nonlinear optical instrumentation, quantitative image analysis, digital pathology, and AI, the results of the INTIMA study will help determine the future role of HHGM within the evolving landscape of renal tumour diagnostics and modern pathology.

## Data Availability

No datasets were generated or analysed during the current study.

## References

[CR1] Aumann K, Hennig G, Schober M et al (2019) Automated analysis of histopathological whole-slide images: where artificial intelligence meets digital pathology. Virchows Arch 475(5):569–579. 10.1007/s00428-019-02625-y

[CR2] Bera K, Schalper KA, Rimm DL, Velcheti V, Madabhushi A (2019) Artificial intelligence in digital pathology — new tools for diagnosis and precision oncology. Nat Rev Clin Oncol 16(11):703–715. 10.1038/s41571-019-0252-y PMC6880861 31399699

[CR3] Campagnola PJ, Loew LM (2003) Second-harmonic imaging microscopy for visualizing biomolecular arrays in cells, tissues and organisms. Nat Biotechnol 21(11):1356–1360. 10.1038/nbt894 14595363

[CR4] Cox G (2011) Biological applications of second harmonic imaging. Biophys Rev 3(3):131–141. 10.1007/s12551-011-0052-9 PMC5425668 28510062

[CR5] Débarre D, Supatto W, Pena AM et al (2006) Imaging lipid bodies in cells and tissues using third-harmonic generation microscopy. Nat Methods 3(1):47–53. 10.1038/nmeth813 16369553

[CR6] Gorrell MD, Wang XM, Levy MT et al (2003) Intrahepatic expression of collagen and fibroblast activation protein (FAP) in hepatitis C virus infection. Adv Exp Med Biol 524:235–243. 10.1007/0-306-47920-6_2 12675244

[CR7] Groot ML, Spies S (2025) Photonic pathways toward real-time tissue assessment. Photoniques 134:30–35. 10.1051/photon/202513430

[CR8] Kuzmin NV, Wesseling P, de Witt Hamer PC et al (2016) Third harmonic generation imaging for fast, label-free pathology of human brain tumors. Biomed Opt Express 7(5):1889–1904. 10.1364/BOE.7.001889 PMC4871089 27231629

[CR9] Orringer DA, Pandian B, Niknafs YS et al (2017) Rapid intraoperative histology of unprocessed surgical specimens via fibre-laser-based stimulated Raman scattering microscopy. Nat Biomed Eng 1:0027. 10.1038/s41551-016-0027 PMC5612414 28955599

[CR10] Provenzano PP, Eliceiri KW, Yan L et al (2008) Nonlinear optical imaging of cellular processes in breast cancer. Microsc Microanal 14(6):532–548. 10.1017/S1431927608080884 PMC5575804 18986607

[CR11] Spies S, Nazarian E, Annavarapu S et al (2025) Multi-observer study on diagnostic accuracy of pediatric renal tumors imaged with higher-harmonic-generation microscopy. Cancers (Basel) 17(10):1693. 10.3390/cancers17101693 PMC12109953 40427190

[CR12] van Huizen LMG, Blokker M, Daniels JMA et al (2025) Rapid on-site histology of lung and pleural biopsies using higher harmonic generation microscopy and artificial intelligence analysis. Mod Pathol 38(1):100633. 10.1016/j.modpat.2024.100633 39424227

